# HER2/neu 655 polymorphism, trastuzumab-induced cardiotoxicity, and survival in HER2-positive breast cancer patients

**DOI:** 10.1007/s12094-024-03512-6

**Published:** 2024-05-21

**Authors:** Isabel Blancas, Marina Linares-Rodríguez, Celia Martín-Bravo, Celia Gómez-Peña, Fernando Rodríguez-Serrano

**Affiliations:** 1 Department of Medicine, University of Granada, Granada, Spain; 2 Section of Medical Oncology, Hospital Universitario Clínico San Cecilio, Granada, Spain; 3 Instituto de Investigación Biosanitaria ibs.GRANADA, Granada, Spain; 4 Biopathology and Regenerative Medicine Institute (IBIMER), Biomedical Research Centre, University of Granada, Avenida del Conocimiento S/N, 18016 Armilla, Granada Spain; 5 Department of Oncology, Costa del Sol Hospital, Marbella, Spain; 6 Department of Pharmacy, Hospital Universitario Clínico San Cecilio, Granada, Spain; 7 Department of Human Anatomy and Embryology, University of Granada, Granada, Spain

**Keywords:** Breast cancer, Trastuzumab, HER2 polymorphism, Cardiotoxicity, Survival

## Abstract

**Purpose:**

HER2 overexpression in breast cancer correlates with poor outcomes. The incorporation of Trastuzumab into the treatment regimen has notably improved patient prognoses. However, cardiotoxicity emerges in approximately 20% of patients treated with the drug. This study aims to investigate the association between the HER2 655 A > G polymorphism, Trastuzumab-induced cardiotoxicity, and patient survival.

**Methods:**

The study involved 88 patients treated with Trastuzumab. Cardiotoxicity, defined as a reduction in left ventricular ejection fraction (LVEF) from baseline or the emergence of clinical signs of congestive heart failure, was identified during treatment follow-up. Genotyping of HER2 655 A > G employed TaqMan SNP technology.

**Results:**

Genotype frequencies of HER2/neu 655 (53 AA, 32 AG, and 3 GG) were consistent with Hardy–Weinberg equilibrium. No significant differences were observed in mean baseline LVEF between patients who developed cardiotoxicity and those who did not. Within these groups, neither AA nor AG genotypes showed an association with changes in mean baseline or reduced LVEF levels. Logistic regression analysis, adjusted for hormonal status and anthracycline treatment, revealed that AG genotype carriers face a significantly higher risk of cardiotoxicity compared to AA carriers (OR = 4.42; p = 0.037). No association was found between the HER2/neu 655 A > G polymorphism and disease-free or overall survival, regardless of whether the data was adjusted for stage or not.

**Conclusion:**

HER2 655 A > G polymorphism is significantly linked to an increased risk of Trastuzumab-induced cardiotoxicity but does not correlate with variations in disease-free survival or overall survival rates.

## Introduction

Human epidermal growth factor receptor 2 (HER2) is a proto-oncogene that encodes a transmembrane protein with tyrosine kinase activity. Approximately 20% of breast cancer patients exhibit overexpression of HER2, which is associated with high rates of recurrence and poor prognoses [1, 2].

Activation of HER2 proteins leads to phosphorylation events, initiating various signaling pathways. These pathways result in the inactivation of proteins that induce apoptosis and up-regulate genes responsible for cellular growth, thereby facilitating tumor cell proliferation and the progression of breast cancer [1].

Trastuzumab, a humanized monoclonal antibody, selectively binds to the extracellular domain of HER2 [3]. Its administration yields an anti-proliferative effect on HER2-positive cells [4] and has been shown to significantly increase response rates in patients with HER2-positive breast cancer, reducing mortality, recurrence, and metastasis rates, and improving overall survival [5–7]. However, a portion of patients develop cardiotoxicity from anti-HER2 therapies [6–9], particularly when Trastuzumab is used in combination with anthracycline-based chemotherapy [4, 5, 10]. The risk of cardiac events induced by Trastuzumab has been reported to range from 3 to 7% when administered alone [11] and as high as 27% when combined with anthracycline [11–13].

The potential of Trastuzumab to cause cardiac complications is linked to its interference with the neuregulin-1 mediated signaling pathway, an essential growth factor for protecting and maintaining cardiac function under oxidative stress. It activates the HER4 receptor, which then forms a dimer with HER2 [14], promoting cardiomyocyte survival and the organization of cardiac contractile structures [15]. Trastuzumab may disrupt the heart ability to protect itself from reactive oxygen species (ROS) and regulate programmed cardiomyocyte death [14, 16].

Given the potential cardiotoxic effects of Trastuzumab, patients necessitate careful monitoring of left ventricular (LV) function, typically evaluated by measuring the LV ejection fraction (LVEF) [17]. However, LVEF may not detect early subtle changes and generally reflects advanced myocyte damage when reduced [18–21].

**Fig. 1:**
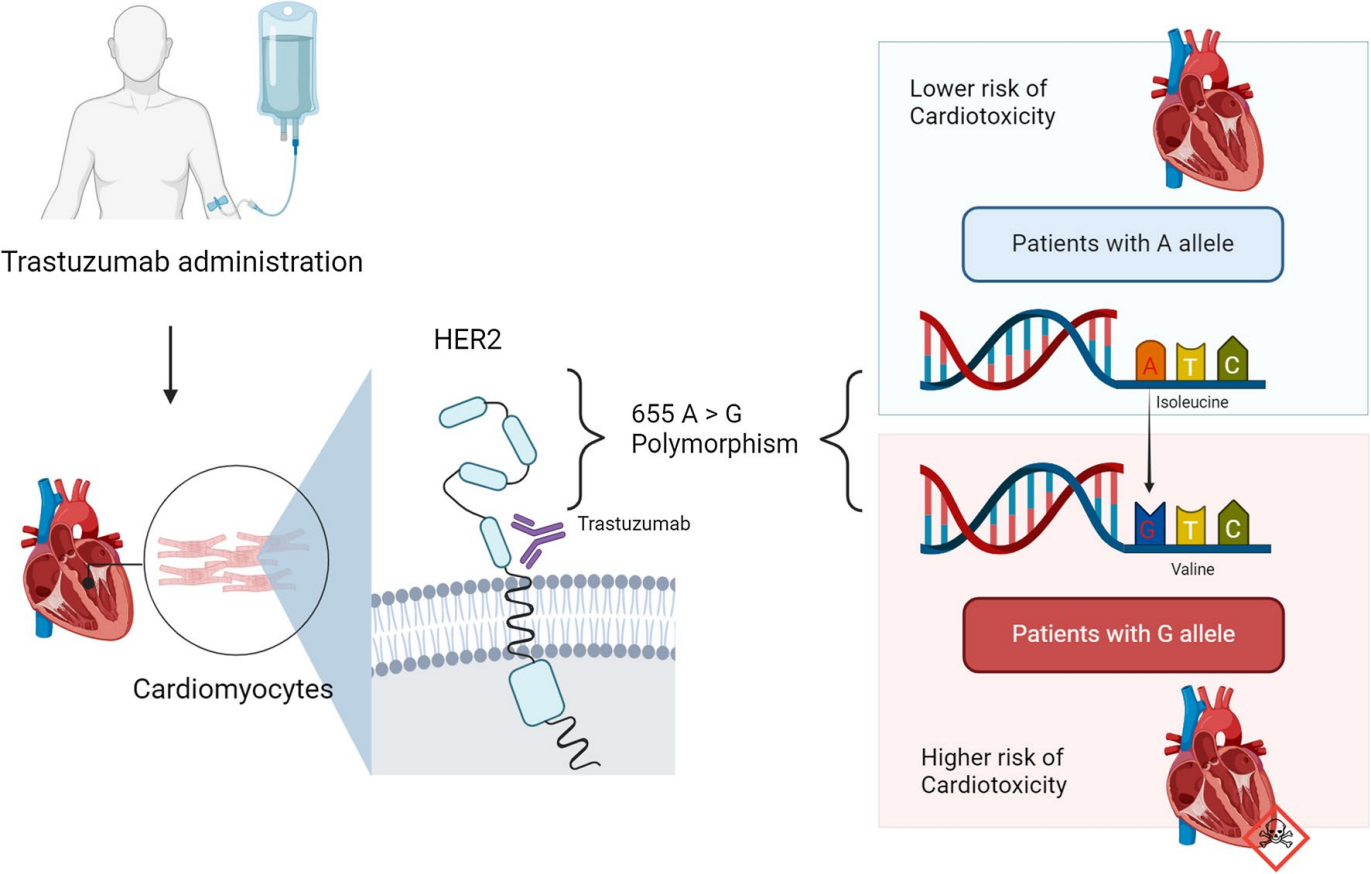
HER2 655 A > G polymorphism and risk of cardiotoxicity during trastuzumab administration

Several genetic polymorphisms in the HER2 gene have been identified [22, 23]. Notably, a polymorphism involves a nucleotide change in the coding region of the transmembrane domain at codon 655, where an adenine (A) is replaced by a guanine (G). This substitution alters the protein structure, replacing isoleucine (Ile) (encoded by ATC) with valine (Val) (encoded by GTC) in the transmembrane domain [24]. The presence of the 655 A variant in the HER2 transmembrane domain may affect the dimerization capability of active HER2 proteins with other HER family members, potentially leading to reduced signal transduction compared to the 655 G variant [23]. Epidemiological studies suggest a weak association between the presence of the 655Val (G) allele and an increased risk of breast cancer in Caucasian populations, while in African women, the Val/Val (G/G) genotype is linked to a higher risk of breast cancer [25]. The 655 A > G polymorphism has also been implicated in influencing the response to Trastuzumab treatment and the likelihood of experiencing cardiac adverse effects [26]. The presence of the G allele may render cardiomyocytes more dependent on HER2 signaling and sensitive to Trastuzumab [27] (Fig. 1).

The objectives of the study include investigating the association between the 655 A > G polymorphism and Trastuzumab-induced cardiotoxicity, as well as its correlation with survival in breast cancer patients. Given the potential of this polymorphism to influence the sensitivity of tumor cells to Trastuzumab, it may have an impact on the survival of breast cancer patients. Understanding the role of the 655 A > G polymorphism in both cardiotoxicity risks and patient survival is crucial for the development of personalized treatment strategies in HER2-positive breast cancer.

## Materials and methods

### Patients and treatment

Eighty-eight HER2-positive early-stage breast cancer patients undergoing Trastuzumab therapy were recruited from San Cecilio University Hospital in Granada (Spain). Informed, written consent was obtained from all participants, and the study received approval from the Provincial Ethical Committee of Granada. Trastuzumab was administered intravenously with an initial dose of 8 mg/kg, followed by a maintenance dose of 6 mg/kg every three weeks, or with an initial dose of 4 mg/kg, followed by a maintenance dose of 2 mg/kg on weekly schedules. Trastuzumab was also administered subcutaneously at a fixed dose of 600 mg. In addition to trastuzumab treatment, fifty-seven patients out of the total received combined therapy with both anthracyclines and taxanes, whereas fifteen patients received exclusive anthracycline therapy, and three patients received exclusive taxane therapy.

### Cardiac toxicity

Cardiac function was assessed in each patient through the measurement of LVEF and clinical examination to detect congestive heart failure or sustained asymptomatic subclinical cardiac toxicity. The patients were assessed through echocardiography or isotopic ventriculography. Patients initially evaluated by one methodology continued their evaluation using the same method throughout the study. LVEF measurements were conducted before the initiation of Trastuzumab treatment and then at 3, 6, 9, and 12 months post the initial administration. Trastuzumab treatment was initiated if LVEF was above 55%, or between 50 and 55% with cardiologist approval. Cardiac toxicity during treatment was defined as any of the following: a decrease of at least 10% from baseline with a resulting LVEF of less than 50% at follow-up, a decrease of 15% relative to baseline LVEF, any decrease resulting in an LVEF of less than 45% at least once during treatment, or clinical manifestation of congestive heart failure [26, 28, 29].

### HER2 655 A > G genotyping

For genotyping, cellular DNA was isolated from saliva samples using standard procedures. The HER2 655 A > G (Ile/Val) (rs1136201) gene promoter single-nucleotide polymorphism was genotyped using the TaqMan SNP Genotyping Assay technology (Life Technologies, Foster City, California, USA). Genotyping analysis was performed using the Applied Biosystems 7500 Fast Real-Time PCR System (Applied Biosystems Inc., Foster City, California, USA) and SDS 2.0.4 software (Applied Biosystems Inc.).

### Statistical analysis

The frequency distribution of HER2 655 A > G polymorphisms was tested for Hardy–Weinberg equilibrium using the chi-squared test. For categorical variables, the chi-squared test or Fisher exact test was employed, depending on the observed frequency in each case. Continuous variables were compared using Student t-test or the Mann–Whitney test as appropriate. The association between cardiac toxicity and genotype was assessed through logistic regression analysis. Odds ratios (ORs) and P-values were calculated, adjusting for confounding variables such as menopausal status and anthracycline use. Survival curves were generated using the Kaplan–Meier method and compared using the log-rank test. Cox proportional hazards analysis was used to determine whether clinical factors (age, menopausal status, anthracycline use or stage) and/or HER2 655 A > G polymorphisms are significantly associated with outcomes. P-values < 0.05 were considered statistically significant. All analyses were conducted using SPSS version 28 (SPSS Inc., Chicago, Illinois, USA).

## Results

### Demographic and clinical features

In this study, 88 HER2-positive breast cancer patients undergoing Trastuzumab treatment were included. The average age of the participants was 50 years (± 11.6), with 46.6% being postmenopausal women. Out of these patients, 19 (21.6%) exhibited cardiotoxicity.

### Association of the HER2 655 (A > G) polymorphism with cardiac toxicity

Genotype frequencies for HER2/neu 655 (53 AA, 32 AG, and 3 GG) were in Hardy–Weinberg equilibrium (p = 0.488). Due to its small size, the GG genotype group was excluded from further analysis. Table 1 indicates that the clinical characteristics between the AA and AG groups were homogeneous, except in the incidence of cardiotoxicity. Logistic regression, adjusted for menopausal status and anthracycline use, revealed an odds ratio for cardiotoxicity of 4.42 (95% CI 1.46–13.40, p = 0.009) in heterozygote AG versus AA patients (Table 2). Consequently, patients with the AG genotype have a significantly higher risk of experiencing cardiotoxicity.

**Table 1:** Demographic and baseline characteristics of patients and tumors

Characteristics	AA (n = 53)	AG (n = 32)	*P*
**Age at diagnosis (years)**			0.716
< 50	26	17	
≥ 50	27	15	
**Menopausal status**			0.890
Premenopause	29	18	
Postmenopause	24	14	
**Stage**			0.744
I	15	7	
II	25	18	
III	10	5	
Unknown	3	2	
**Histologic subtype**			1.000
Invasive ductal	49	30	
Invasive lobular	2	1	
Others	2	1	
**Estrogen receptor**			0.367
Positive	35	18	
Negative	18	14	
**Progesterone receptor**			0.446
Positive	31	16	
Negative	22	16	
**Ki-67**			0.549
Yes	28	19	
No	6	6	
Unknown	19	7	
**Treatment**			0.230
Neoadjuvant	19	12	
Adjuvant	27	15	
Both	4	0	
None	3	5	
**Anthracyclines**			0.576
Yes	44	25	
No	9	7	
**Radiotherapy**			0.823
Yes	42	26	
No	11	6	
**Cardiotoxicity**			**0.009**
Yes	7	12	
No	46	20	

Bold value indicates statistical significance

**Table 2:** HER2-655 A ˃ G genetic variant and cardiotoxicity in breast cancer patients treated with Trastuzumab*

Genotype	Cardiotoxicity [N (%)]	Non-cardiotoxicity [N (%)]	OR (95% CI)	*P-value*
AA	7 (13.2)	46 (86.8)	1.00 (Reference)	
AG	12 (37.5)	20 (62.5)	**4.42 (1.46–13.40)**	**0.009**

Bold values indicate statistical significance

^*^Logistic regression adjusted by menopausal status and anthracyclines (adjuvant and neoadjuvant)

### Association of genotype, LVEF, and cardiac toxicity

Table 3 presents the relationship between the HER2-655 A > G genetic variant, cardiotoxicity, baseline LVEF, and the lowest LVEF recorded during patient follow-up, assessed by echocardiography or isotopic ventriculography. Among the 19 patients who developed cardiotoxicity, 12 had the AG genotype, with a mean baseline LVEF of 60.2. In contrast, of the 66 patients who did not experience cardiotoxicity, 46 were of the AA genotype. No significant differences were observed in the HER2-655 genotype related to LVEF in either the cardiotoxicity or non-cardiotoxicity groups. Moreover, no significant differences were found in mean baseline LVEF when comparing patients who developed cardiotoxicity to those who did not.

**Table 3:** Influence of HER2-655 A ˃ G genetic variant on LVEF in cardiotoxicity or non-cardiotoxicity patients

		LVEF baseline			Lower LVEF		
Genotype	N	Mean	IC95%	P	Mean	IC95%	P
**Cardiotoxicity**							
AA	7	63.4	59.3–67.6	NS	52.1	48.5–55.8	NS
AG	12	60.2	57.0–63.3		50.5	47.7–53.3	
**Non-cardiotoxicity**							
AA	46	61.6	59.9–63.2	NS	58.3	56.8–59.7	NS
AG	20	60.9	58.5–63.4		58.9	56.7–61.0	

Left ventricular ejecdtion fraction (*LVEF*)

### Influence of HER2 655 A > G polymorphism on disease-free survival and overall survival

**Fig. 2:**
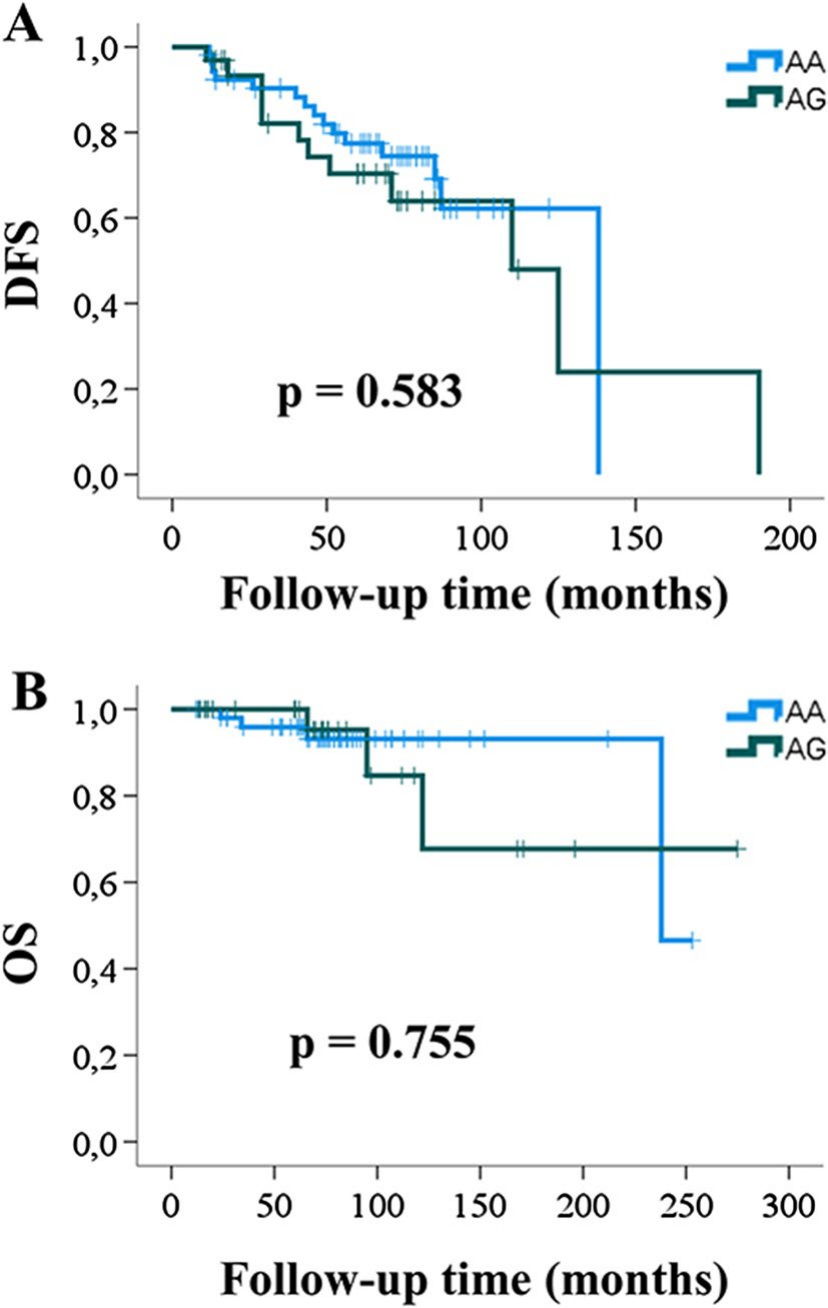
Kaplan–Meier survival analysis of breast cancer patients treated with Trastuzumab in relation to HER2-655 A > G genetic polymorphism (AA vs AG)

The influence of the HER2 A > G 655 polymorphism on the survival of HER2-positive breast cancer patients was evaluated using Kaplan–Meier analysis, with a mean follow-up duration of 83.1 months. No statistical relationship was observed between disease-free survival (DFS) or overall survival (OS) and the genotype, regardless of whether the data were adjusted for stage (Table 4) or not (Fig. 2). Cox proportional hazards analysis was employed to ascertain whether clinical factors (age, menopausal status, anthracycline use or stage) and/or the HER2 655 A > G polymorphism significantly influenced outcomes (Table 5). It was found that only stage and menopausal status were associated with DFS, while stage and age correlated with OS. Notably, the AA/AG polymorphism did not significantly affect survival outcomes.

**Table 4:** Survival data according to stage and HER2 655 genotype

Kaplan–Meier			DFS					OS				
					IC 95%					IC 95%		
Stage	Genotype	N	Relapse	Mean	Inf	Sup	Log Rank	Exitus	Mean	Inf	Sup	Log Rank
I	AA	15	1	95.0	87.4	102.6	0.960	0	–	–	–	0.959
	AG	7	1	110.0	110.0	110.0		0	–	–	–	
II	AA	25	6	115.3	96.5	134.1		0	–	–	–	
	AG	18	6	120.7	74.0	167.3		1	–	–	–	
III	AA	10	7	50.8	30.4	71.2		4	179.4	116.9	241.8	
	AG	5	3	51.2	9.3	33.0		1	89.3	76.1	102.4	

Disease−free survival (*DFS*), Overall survival (*OS*)

**Table 5:** Relation between patient features and disease-free survival and overall survival

Cox regression	DFS		OS	
	HR	*P-value*	HR	*P-value*
**Age:** < 50 vs ≥ 50 (R.)	4.13	0.137	0.006	**0.035**
**Menopausal status**: Pre vs Post (R.)	0.12	**0.027**	7.24	0.319
**Anthracyclines:** Yes vs No (R.)	1.57	0.491	4.52	0.303
**Stage:** I vs III (R.)	0.05	** < 0.001**	0.00	0.971
**Stage:** II vs III (R.)	0.18	**0.001**	0.008	**0.007**
**Polymorphism:** AA vs AG (R.)	0.85	0.713	1.49	0.725

Bold values indicate statistical significance

*R*. Reference group (HR = 1) in the multivariable cox regression analysis

## Discussion

Trastuzumab treatment has significantly improved outcomes for patients with HER2-positive breast cancer. However, a subset of these patients develops cardiac toxicity [5, 6]. Given the risks of adverse effects of anti-HER2 therapy, predicting which patients might develop cardiac toxicity is crucial for preventing cardiac complications. Our study was conducted in a cohort of Spanish patients of Caucasian descent, and the observed genotype frequencies were consistent with Hardy–Weinberg equilibrium. To the best of our knowledge, no study has indicated significant divergences in allelic or genotypic frequencies associated with the HER2 655 A > G polymorphism within the Spanish population. However, relevant data have been documented in other populations. A study encompassing premenopausal women of diverse ethnic backgrounds, including Ghanaian, Caucasian, African American, Kenyan, Filipino, Saudi Arabian, and Sudanese, revealed that the homozygous G/G genotype exhibited a higher prevalence among Caucasians (5.4%) and African Americans (5.4%) compared to Saudi subjects (2.0%) and Chinese subjects (0.3%). It is noteworthy that this genotype was not detected in any of the three studied continental African populations (Ghanaian, Kenyan, Sudanese). Additionally, no statistically significant differences were evidenced in the frequency of the HER-2 allele between African Americans and Caucasians (P > 0.49) [30] In another study conducted on the Greek population, which included 45 women diagnosed with breast cancer (32 Christians and 13 Muslims) and 56 control cases (43 Christians and 13 Muslims), it was observed that the frequency of the G allele and genotypes containing G was notably higher among Muslims compared to Christians (p = 0.020 and p = 0.008, respectively). Within the Greek Christian population, the risk of breast cancer demonstrated a fivefold increase with the Val/Val genotype and a 3.1-fold increase with the Ile/Val or Val/Val genotypes (95% CI, 1.3–18.4; p = 0.017 and OR, 3.1; 95% CI, 1.2–8.3; p = 0.025; respectively) when compared to the homozygous A/A genotype [31].

Our results indicate that the HER2 655 A > G polymorphism is a genetic predictor of Trastuzumab-induced cardiotoxicity in HER2-positive breast cancer patients. In our study, cardiotoxicity was assessed based on LVEF, which was measured using either echocardiography or isotopic ventriculography. Patients with the HER2-AG genotype are at a higher risk for cardiotoxicity. Other studies examining the impact of this polymorphism in HER2-positive breast cancer patients on cardiotoxicity under Trastuzumab therapy have yielded varying results (Table 6).

**Table 6:** Relationship between genotypes of HER2 655 A > G polymorphism and cardiotoxicity in patients treated with trastuzumab in different studies

	Cases	Cardiotoxicity	Non-Cardiotoxicity	Country	Years
**Blancas** **et al***.*** (present study)**				Spain	2024
AA	53	7	46		
AG	32	12	20		
GG	3	1	2		
Total	88	20	68		
**Beauclair et al.**				France	2007
AA	36	0	36		
AG	21	5	16		
GG	4	0	4		
Total	61	5	56		
**Lemieux et a**l.				Canada	2013
AA	52	4	48		
AG	18	6	12		
GG	3	0	3		
Total	73	10	63		
**Roca et al.**				France	2013
AA	79	4	75		
AG	48	9	39		
GG	5	0	5		
Total	132	13	119		
**Gómez-Peña et al.**				Spain	2015
AA	50	6	44		
AG	26	9	17		
GG	2	0	2		
Total	78	15	63		
**Tan et al.**				China	2020
AA	64	13	51		
AG	24	11	13		
GG	3	2	1		
Total	91	26	65		

In one study exploring the HER2 655 A > G polymorphism, a potential link was investigated between this polymorphism and the occurrence of cardiotoxicity. Cardiotoxicity was assessed through a multiple-gated acquisition scan conducted every 3 months. Among patients treated with Trastuzumab, cardiotoxicity was identified in 8.2% of those with the AG genotype. It was hypothesized that cells expressing the G isoform might experience increased proliferation rates and higher susceptibility to Trastuzumab. Consequently, it was suggested that the presence of the G allele could lead to a dependency of cardiomyocytes on HER2 signaling, resulting in heightened sensitivity to Trastuzumab [27].

A separate investigation involving 78 HER2-positive patients treated with Trastuzumab observed cardiotoxicity in 19.23% of cases. This rate aligns closely with the cardiotoxicity rate observed in our study. Of these, 40% had the AA genotype, while 60% had the AG genotype, a statistically significant difference. The findings concluded that patients with the 655 A > G polymorphism were at an increased risk of developing cardiotoxicity during Trastuzumab treatment [26]. Further analysis focused on the polymorphism in another group of patients found that 71% of patients had the A/A genotype, 25% were A/G, and 4% were G/G. 33% of patients with the A/G genotype exhibited cardiac toxicity, compared to only 8% of patients with the A/A genotype. These differences were statistically significant, supporting the idea that the HER2 A/G genotype could be considered a risk factor for cardiotoxicity during Trastuzumab treatment [32]. In another study conducted in China with 91 patients treated with anthracycline and trastuzumab combined adjuvant chemotherapy, 28.6% developed cardiotoxicity, while 71.4% did not. The patients were assessed at M0, M3, M6, M9, M12, and M15 using an ultrasonic cardiogram, and LVEF was identified through echocardiography. The incidence of cardiotoxicity exhibited a notable increase in patients harboring AG/GG genotypes at the HER2 codon 655, as opposed to individuals with the AA genotype at the same codon (P = 0.007) [33].

Another study, conducted in 132 patients, examined various polymorphisms, including HER2 655 A > G, noted that patients with the G allele in their genotype (A/G + G/G) had a significantly higher number of cardiac alterations compared to those with the A/A genotype. LVEF was assessed through multiple-gated acquisition scanning or echocardiography. Measurements were taken before the administration of trastuzumab and at 1, 6, 9, 12, 16, and 20 months after the initial trastuzumab administration, and subsequently at the 5-year follow-up [34]. A meta-analysis consolidating data from several studies [26, 27, 32, 34] supported the conclusion of a potential link between Trastuzumab-induced cardiotoxicity and the HER2-AG genetic variant. The absence of cardiotoxicity in GG patients was noted to be due to the low representation of this genotype [26]. Similarly, in our study, only 3 patients with the GG genotype were present, which is insufficient for statistical analysis. However, interestingly, two of these patients relapsed, and one died. While the data is limited, this observation could be intriguing for future studies.

However, other studies have not found an association between cardiotoxicity and the genetic variant of codon 655 [35, 36]. In a study with 140 patients treated with Trastuzumab, 29 experienced cardiotoxicity. The AA variant was present in 68% of the patients, AG in 29%, and GG in 3%. No significant relationship was observed between the codon 655 polymorphism and cardiotoxicity (p = 0.96). However, a significant connection was found between cardiotoxicity and codon 1170 when the genotype was Pro/Pro, compared to Pro/Ala or Ala/Ala cases [36].

Our results show a correlation between the HER2 A > G polymorphism and Trastuzumab-induced cardiotoxicity. The model proposed by Beauclair et al*.* could explain this phenomenon. They found a difference in tumor induction between the G and A alleles and discovered that G-carrying clones exhibited higher sensitivity to Trastuzumab. Experimental results indicate that cells with the G allele have enhanced growth capability and increased sensitivity to Trastuzumab, suggesting that the presence of the G allele could result in cardiomyocytes particularly dependent on HER2 signaling and, therefore, highly sensitive to Trastuzumab [27]. An alternative genetic model linked cardiotoxicity to the HER2 A > G genotype, proposing that patients with both AA and GG alleles have a similar risk of not experiencing cardiotoxicity compared to heterozygous (AG) patients. The HER2 655 A > G polymorphism would cause alterations in the balance between the active and inactive states of the HER2 protein in the body [23].

In our study, we also analyzed the relationship between different HER2 655 genotypes and disease stages (I, II, or III) in the context of DFS and OS. Our findings did not reveal any significant statistical associations, indicating that the A > G polymorphism may not impact patient survival in these cases. To the best of our knowledge, little is known about the potential association between the HER2 655 A > G genotype and DFS in HER2-positive status.

In contrast, another study examining 71 patients with HER2-positive breast cancer treated with Trastuzumab found that individuals with AG or GG genotypes had poorer DFS than those with the AA genotype (P = 0.01) [37]. A study involving HER2-positive patients in an early-stage undergoing adjuvant chemotherapy and/or endocrine therapy without Trastuzumab treatment reported that those with AG or GG genotypes had significantly poorer DFS compared to the AA genotype (P = 0.037). However, among 212 patients treated with chemotherapy and Trastuzumab, the AG or GG genotypes were associated with better DFS than the AA genotype (5-year DFS, P = 0.008), suggesting that the HER2 655 A > G polymorphism influences the HER2 gene function in HER2-positive breast cancers, with the G variant carriers having an aggressive phenotype but showing sensitivity to Trastuzumab. The chemo backbone utilized by 212 patients in this study was adjuvant treatment with trastuzumab combined with adjuvant chemotherapy, which included an anthracycline-based regimen (n = 23), an anthracycline-based regimen followed by taxanes (n = 131), or a taxane-based regimen (n = 58) [38]. In the patients participating in our study, had early-stage breast cancer and all of them were treated with trastuzumab. Additionally, 57 patients were treated with anthracyclines and taxanes, 15 patients were treated with anthracyclines alone and 3 patients were treated with taxanes alone. Similarly to Han et al., we did not find significant differences regarding DFS or OS in patients treated with trastuzumab. The reason for this outcome could be, as indicated by Han et al., because this drug counteracts the negative effect of carrying the G variant. Consistently to our study and Han et al. study, other studies, found no significant differences in DFS were observed between genotypes [27, 39].

Finally, different clinical characteristics with respect to DFS and OS were analyzed. Having a more advanced stage was significant in relation to DFS (P < 0.001) and also for OS (P = 0.007) when comparing stage III with stage II, as found in other studies [38, 40, 41]. This study also found significance for DFS (P = 0.027), indicating that postmenopausal patients have a higher risk of progression, as seen in Goss et al., where premenopausal patients treated with letrozole had a higher DFS (96.5 vs 93.9%) [42]. On the other hand, age, specifically being over 50 years old, was significant for OS (P = 0.035), mirroring other studies where age was a significant factor in OS [43, 44]. However, the HER2 655 A > G polymorphism (AA vs AG) was not associated with survival.

One limitation of our study is the small number of patients, especially in the GG genotype group, where only three patients were included. Notably, among these, two patients experienced a relapse, and one died. While this limited sample size constrains the robustness of our analysis, these observations suggest potential avenues for further research. This study significantly contributes to the ongoing debate about the cardiotoxic potential of trastuzumab in patients with the HER2 655 A > G polymorphism. By offering valuable data, our research clarifies the relationship between this specific polymorphism and the adverse effects of trastuzumab. The novelty of our work lies in its comprehensive analysis of the influence of the 655 A > G polymorphism on trastuzumab response and its integration with survival data in relation to clinical characteristics. This approach enhances our understanding of the polymorphism impact, underscoring the need for personalized treatment strategies in HER2-positive breast cancer.

## Conclusion

Our study supports the role of the HER2 655 A > G polymorphism as a potential genetic risk factor for Trastuzumab-induced cardiotoxicity in patients with HER2-positive breast cancer. However, this polymorphism was not associated with survival. Further research, incorporating a larger patient cohort that could lead to an increase in the number of GG genotype patients, is required to validate the genetic model. This could lead to the establishment of a robust pharmacogenetic marker that could assist physicians in managing Trastuzumab-induced cardiotoxicity more effectively.

## Data Availability

The datasets generated and analyzed during the current study are available from the corresponding authors on reasonable request.

## Abbreviations

DFS: Disease-free survival
HER2: Human epidermal growth factor receptor 2
LV: Left ventricle
LVEF: Left ventricular ejection fraction
OS: Overall survival
